# Inflammasome and toll-like receptor signaling in human monocytes after successful cardiopulmonary resuscitation

**DOI:** 10.1186/s13054-016-1340-3

**Published:** 2016-06-04

**Authors:** Alexander Asmussen, Katrin Fink, Hans-Jörg Busch, Thomas Helbing, Natascha Bourgeois, Christoph Bode, Sebastian Grundmann

**Affiliations:** Department of Cardiology and Angiology I, Heart Center Freiburg University, Hugstetter Straße 55, Freiburg im Breisgau, 79106 Germany; Department of Emergency Medicine, University Medical Center Freiburg, Sir-Hans-A.-Krebs-Straße, Freiburg im Breisgau, 79106 Germany

**Keywords:** Post-cardiac arrest syndrome, Cardiopulmonary resuscitation, Toll-like receptor, Inflammasome, Endotoxin tolerance, Monocyte

## Abstract

**Background:**

Whole body ischemia-reperfusion injury (IRI) after cardiopulmonary resuscitation (CPR) induces a generalized inflammatory response which contributes to the development of post-cardiac arrest syndrome (PCAS). Recently, pattern recognition receptors (PRRs), such as toll-like receptors (TLRs) and inflammasomes, have been shown to mediate the inflammatory response in IRI. In this study we investigated monocyte PRR signaling and function in PCAS.

**Methods:**

Blood samples were drawn in the first 12 hours, and at 24 and 48 hours following return of spontaneous circulation in 51 survivors after cardiac arrest. Monocyte mRNA levels of TLR2, TLR4, interleukin-1 receptor-associated kinase (IRAK)3, IRAK4, NLR family pyrin domain containing (NLRP)1, NLRP3, AIM2, PYCARD, CASP1, and IL1B were determined by real-time quantitative PCR. Ex vivo cytokine production in response to stimulation with TLR ligands Pam_3_CSK_4_ and lipopolysaccharide (LPS) was assessed in both whole blood and monocyte culture assays. Ex vivo cytokine production of peripheral blood mononuclear cells (PBMCs) from a healthy volunteer in response to stimulation with patients’ sera with or without LPS was assessed. The results were compared to 19 hemodynamically stable patients with coronary artery disease.

**Results:**

Monocyte TLR2, TLR4, IRAK3, IRAK4, NLRP3, PYCARD and IL1B were initially upregulated in patients following cardiac arrest. The NLRP1 and AIM2 inflammasomes were downregulated in resuscitated patients. There was a significant positive correlation between TLR2, TLR4, IRAK3 and IRAK4 expression and the degree of ischemia as assessed by serum lactate levels and the time until return of spontaneous circulation. Nonsurvivors at 30 days had significantly lower mRNA levels of TLR2, IRAK3, IRAK4, NLRP3 and CASP1 in the late phase following cardiac arrest. We observed reduced proinflammatory cytokine release in response to both TLR2 and TLR4 activation in whole blood and monocyte culture assays in patients after CPR. Sera from resuscitated patients attenuated the inflammatory response in cultured PBMCs after co-stimulation with LPS.

**Conclusions:**

Successful resuscitation from cardiac arrest results in changes in monocyte pattern recognition receptor signaling pathways, which may contribute to the post-cardiac arrest syndrome.

**Trial registration:**

The trial was registered in the German Clinical Trials Register (DRKS00009684) on 27/11/2015.

**Electronic supplementary material:**

The online version of this article (doi:10.1186/s13054-016-1340-3) contains supplementary material, which is available to authorized users.

## Background

The annual incidence of sudden cardiac arrest ranges between 50 and 100 per 100,000 in the general population in North America and Europe. A recent registry study for out-of-hospital cardiac arrest (OHCA) shows that although a return of spontaneous circulation (ROSC) is obtained in 34.4 %, the prognosis of patients suffering sudden cardiac arrest still remains poor, with an overall survival to hospital discharge rate of 9.6 % [1]. This high mortality rate in patients who initially achieve ROSC can be attributed to a unique pathophysiological condition involving multiple organs known as post-cardiac arrest syndrome (PCAS) [2, 3]. PCAS is characterized by its four major clinical components, namely (1) anoxic brain injury, (2) myocardial dysfunction, (3) systemic ischemia-reperfusion response, and (4) the persistent precipitating pathology [3]. On a pathophysiological level, the initial tissue injury during sudden whole-body ischemia is thought to be aggravated during reperfusion through cardiopulmonary resuscitation and finally by ROSC, resulting in the generation of reactive oxygen species and thereby inducing oxidative stress [4–6]. These events lead to the induction of a systemic inflammatory response with neutrophil activation [7], elevation of plasma cytokines [8] and severe endothelial injury [9–11]. These deleterious pathological processes contribute to microcirculatory disorder [12–14] and vascular leakage [14, 15] and may finally result in a clinical condition comparable to septic shock [8, 16]. However, up to this day, the only causative treatment in post-cardiac arrest care remains therapeutic hypothermia [17].

The aim of this study was to investigate the potential involvement of the innate immune system as a potential modulating factor in the inflammatory response following cardiac arrest. While its important role is well documented in sepsis [18], trauma [19], and tissue damage after ischemia-reperfusion injury (IRI) in specific organs [20], little is known about the contribution of innate immunity to the systemic inflammatory response syndrome after cardiac arrest. As one of the evolutionary oldest barriers against pathogen invasion, the innate immune system recognizes pathogen-associated molecular patterns (PAMPs) via germline-encoded pattern-recognition receptors (PRRs), which lead to an antimicrobial response. Toll-like receptors (TLRs), members of membrane-bound PRRs, and the inflammasomes, which are PRRs located in the cytoplasm, therefore play a pivotal role in the first line of host defense against pathogens by inducing proinflammatory cytokines like interleukin-1 beta (IL-1β) and tumor necrosis factor alpha (TNFα) [21, 22]. It is now evident that these PRRs also play a crucial role in conditions of sterile inflammation, like in IRI, as these receptors also recognize a heterogeneous group of endogenous alarm signals. These danger-associated molecular patterns (DAMPs) [23], such as heat-shock proteins, uric acid, genomic double-stranded DNA, and components of the extracellular matrix, are cell-derived molecules that are released by injured or distressed cells and tissue [24] and can contribute to inflammation via activation of PRRs [25, 26].

In the current study we therefore investigated the involvement of the toll-like receptors and the inflammasome in the systemic inflammatory condition following survived cardiac arrest. Our working hypothesis was that global ischemia-reperfusion injury, induced by circulatory arrest and cardiopulmonary resuscitation, results in the release of DAMPs which activate PRRs. We further hypothesized that this activation results in an expressional change of these receptors at the mRNA level, which alters the response of these PRRs to subsequent stimuli.

## Methods

### Patient recruitment

The study was approved by the ethics committee of the University Medical Center Freiburg (approval number 328/09) and conforms to the declaration of Helsinki. The trial was registered in the German Clinical Trials Register (DRKS00009684). We prospectively enrolled 55 patients who had undergone successful cardiopulmonary resuscitation (CPR), and were admitted to our intensive care unit at the University Hospital of Freiburg, Germany. The patients’ next of kin were informed about the study details. Informed consent was obtained retrospectively from patients who survived to hospital discharge with a good neurological outcome. A total of 20 patients with both stable and unstable coronary artery disease (CAD), but without acute myocardial infarction, were included in this study as control subjects, because the comorbidities and pharmacological and interventional treatment of patients with sudden cardiac arrest is most closely reflected by this group of patients. Written informed consent was obtained from all patients in the control group. Four patients (cases) and one control subject were retrospectively excluded from the study because of violation of the exclusion criteria, which was not evident at the time of study enrollment.

### Inclusion and exclusion criteria

Patients older than 18 years with either in-hospital cardiac arrest (IHCA) or out-of-hospital cardiac arrest (OHCA) due to any cause, who received cardiopulmonary resuscitation for longer than 5 minutes (including downtime before the beginning of CPR) were included in this study. Patients with preexisting acute or chronic inflammatory or infectious disease, and patients taking immunosuppressive medication were excluded from this study, as in these patients a modulation of the monocyte inflammasome or TLR signaling can be expected [22, 27]. Furthermore, patients with apparent multiple organ dysfunction syndrome prior to cardiac arrest were excluded from this study [28].

### Sample collection

Blood samples were drawn from resuscitated patients via an arterial line within the first 12 h after admission to our hospital, and after 24 and 48 h, respectively. In the control group, a single blood specimen was collected by sterile venipuncture with a 21-gauge butterfly needle. Samples were drawn slowly and immediately processed.

### Monocyte purification

Peripheral blood mononuclear cells (PBMCs) were purified from fresh citrated blood by Biocoll-1.077 density gradient separation (Biochrom, Berlin, Germany) at 460 × g for 30 minutes at room temperature. The mononuclear cell layer was removed and washed two times in cold Dulbecco’s phosphate-buffered saline (DPBS) (Life Technologies, Carlsbad, CA, USA), w/o CaCl_2_ and MgCl_2_, with 2 mM EDTA, by centrifugation at 200 × g for 12 minutes at 4 °C. Monocytes were isolated by negative selection with the Monocyte Isolation Kit II (Miltenyi Biotech, Bergisch-Gladbach, Germany) according to the manufacturer’s instructions. Monocyte purification success was verified by flow cytometry analysis.

### RNA extraction

Total RNA was extracted by phenol/guanidine-based lysis of monocyte samples and silica membrane-based purification with the miRNeasy Mini Kit (Qiagen, Venlo, Netherlands) according to the manufacturer’s protocol. RNA quality and quantity was assessed by Nanodrop spectrophotometer (Thermo Fisher Scientific, Waltham, MA, USA). An absorption coefficient at 260 nm/280 nm from 1.8 to 2.0 was considered as pure RNA and led to further processing of the RNA specimen.

### cDNA synthesis and quantitative real-time PCR

RNA was reverse transcribed with the Transcriptor First Strand cDNA Synthesis Kit (Roche, Basel, Switzerland). The converted cDNA was used for quantitative real-time polymerase chain reaction (qPCR) analysis with the Light Cycler 480 SYBR Green Master I Kit on a Light Cycler 480 Instrument II (Roche, Basel, Switzerland). Primer-pairs were designed with Beacon Designer software (Premier Biosoft, Palo Alto, CA, USA) and are listed in a supplementary table (Additional file 1). Intron-spanning primer-pairs were preferred over intron-flanking primer-pairs. Primer-pair efficiency was determined using a standard curve dilution method. A primer-pair efficiency of 90–110 % was accepted. Relative quantification was used to assess the gene expression of selected genes linked to monocyte TLR and inflammasome signaling. Gene expression was normalized to the two reference genes RNA polymerase 2 (POL2RA) and Beta-2 microglobulin (β2M). POL2RA has been shown to be constantly expressed over multiple mammalian cell lines [29], whereas β2M has been shown to be steadily expressed in activated monocytes after stimulation with lipopolysaccharide (LPS) [30]. Relative copy numbers (RCN) of the selected genes were calculated using the equation:

$$ \mathrm{R}\mathrm{C}\mathrm{N} = {\mathrm{E}}^{-\varDelta \mathrm{C}\mathrm{t}} $$

where E is the primer efficiency of the target gene and ∆Ct is the difference of the threshold cycles of the target gene and the geometric mean of the threshold cycles of the two reference genes.

### Stimulation of whole blood samples, cultured monocytes and PBMCs

NH4-heparinized whole blood (100 μl) was stimulated in sterile 96-well plates with 100 μl TLR4 ligand LPS from *Escherichia coli* 055:B5 (Sigma, Missouri, USA) at a final concentration of 10 ng/ml and 100 μl of the synthetic TLR2 ligand Pam_3_CSK_4_ (Merck Millipore, Darmstadt, Germany) at a final concentration of 500 ng/ml as previously described [31]. For stimulation of isolated monocytes, 10^6^ purified monocytes were resuspended in 900 μl Roswell Park Memorial Institute (RPMI)-1640 medium supplemented with 2 mM L-glutamine, 1 % non-essential amino acid solution, 200 U/ml penicillin, 200 μg/ml streptomycin, and 10 % fetal calf serum in sterile 12-well plates. Monocytes were stimulated with 100 μl LPS for a final concentration of 10 ng/ml. PBMCs were isolated from a healthy control. For stimulation of PBMCs, 0.5 × 10^6^ PBMCs were resuspended in 400 μl RPMI-1640 medium supplemented with 2 mM L-glutamine, 1 % non-essential amino acid solution, 200 U/ml penicillin, 200 μg/ml streptomycin, and incubated with 100 μl serum at a final concentration of 20 % from either resuscitated patients or patients with CAD. Additionally, 0.5 × 10^6^ PBMCs were co-stimulated with 20 % patient serum and 10 ng/ml LPS in the previously described cell culture medium. Whole blood, monocyte, and PBMC cultures were incubated for 12 h at 37 °C and 5 % CO_2_. The culture supernatant was stored at −20 °C for further analysis.

TNF-α was determined in TLR2 ligand-activated whole blood supernatants using an enzyme-linked immunosorbent assay (ELISA) (PeliKine compact, Sanquin Reagents, Amsterdam, Netherlands). IL-1β was determined in TLR4 ligand-stimulated whole blood, monocyte, and PBMC culture supernatants (RayBio Human IL-1β ELISA, RayBiotech, Norcross, GA, USA) according to the manufacturer’s protocol. The resulting cytokine concentration was standardized to the patient’s white blood count in whole blood culture supernatants.

### Statistics

Statistical analysis was performed using SPSS 21 (IBM, Armonk, NY, USA). Gaussian distribution was verified by visualization of the respective histograms, the Shapiro-Wilk test, and a calculation of the *z* score of skewness and kurtosis. A *z* score of 1.96 was considered as statistically not significant and a normal distribution was assumed [32]. The assumption of homogeneity of variances was verified by the nonparametric Levene test [33]. Fisher’s exact test was used to compare categorical variables. Normally distributed unpaired data on an interval scale consisting of multiple groups were analyzed with one-way analysis of variance (ANOVA) and post-hoc analysis with all-pairwise comparison. Non-normally distributed unpaired data on an interval scale consisting of two groups were analyzed using the Mann–Whitney *U* test. Non-normally distributed unpaired data on an interval scale consisting of multiple groups were analyzed with Kruskal-Wallis test and post-hoc analysis using the Dunn-Bonferroni approach. Correlation between selected variables was estimated by Spearman's rank correlation. Statistical significance was defined as a two-tailed *p* value <0.05. Continuous variables are reported as mean value ± standard deviation (SD). Bar graphs illustrate the mean value, with the error bars indicating the SD.

## Results

### Patient characteristics

A total of 51 patients who had undergone cardiopulmonary resuscitation (CPR group) and 19 patients with CAD were included in this study. The majority of the study population was male. Mean age at the time of the investigation did not differ significantly between the two groups (66.5 ± 11.5 in the resuscitation group vs. 68.9 ± 11.6 in the CAD group; *p* = 0.44). Of the resuscitated patients, 67 % had significant CAD vs. 100 % in the CAD group (*p* = 0.003). Although more patients in the CPR group underwent coronary angiography prior to (<12 h before) study enrollment (CPR group 76 % vs. CAD group 42 %; *p* = 0.01), there was no difference between the two groups in the resulting coronary revascularization through percutaneous coronary intervention (PCI) (CPR 45 % vs. CAD 42 %; *p* = 1.0) (Table 1).

**Table 1:** Patient characteristics

	CPR group (*n* = 51)	CAD group (*n* = 19)	*P* value
Age (years)	66.49 ± 11.53	68.89 ± 11.60	0.441
Gender male:female	39:12	15:4	1.0
CPR scene			
OHCA	41 (80 %)	N/A	
IHCA	10 (20 %)	N/A	
Etiology of cardiac arrest			
Cardiac	32 (63 %)	N/A	
Non-cardiac	13 (25 %)	N/A	
Unknown	6 (12 %)	N/A	
Initial rhythm			
VT/VF	29 (57 %)	N/A	
Asystole/PEA	22 (43 %)	N/A	
Time from collapse to CPR (minutes)	2.43 ± 3.88	N/A	
Time from collapse to ROSC (minutes)	29.84 ± 19.11	N/A	
Interventions			
Therapeutic hypothermia performed	50 (98 %)	N/A	
Coronary angiography <12 h prior to study enrollment	39 (76 %)	8 (42 %)	0.01
PCI <12 h prior to study enrollment	23 (45 %)	8 (42 %)	1.0
Consecutive organ failure			
Acute heart failure	19 (37 %)	0 (0 %)	0.002
Acute respiratory failure	7 (14 %)	0 (0 %)	0.177
Acute liver failure	0 (0 %)	0 (0 %)	N/A
Acute renal failure	16 (31 %)	0 (0 %)	0.004
Sequential organ failure assessment (SOFA) score			
Day 1 after ROSC	10.53 ± 1.75	N/A	
Day 2 after ROSC	10.83 ± 1.63	N/A	
Day 3 after ROSC	10.85 ± 1.83	N/A	
Medical history			
Coronary artery disease	34 (67 %)	19 (100 %)	0.003
Peripheral artery disease	3 (6 %)	1 (5 %)	1.0
Chronic heart failure	9 (18 %)	3 (16 %)	1.0
Pulmonary hypertension	6 (12 %)	0 (0 %)	0.180
Chronic lung disease	14 (27 %)	1 (5 %)	0.052
Chronic liver disease	0 (0 %)	0 (0 %)	N/A
Chronic kidney disease	6 (12 %)	5 (26 %)	0.155
Cardiovascular risk factors			
Hypertension	29 (57 %)	15 (79 %)	0.104
Diabetes	13 (25 %)	6 (32 %)	0.763
Dyslipidemia	16 (31 %)	13 (68 %)	0.007
Smoking	19 (37 %)	12 (63 %)	0.063
Overweight	14 (27 %)	7 (37 %)	0.559

Acute heart failure was defined by clinical signs of cardiac decompensation or cardiogenic shock. Acute renal failure was defined as an increase in serum creatinine ≥0.3 mg/dl or ≥1.5-fold increase from baseline creatinine within the first 48 h. Acute liver failure was defined as an increase in total bilirubin serum levels and an increase in the international normalized ratio (INR) value above the normal values of our central laboratory. Acute respiratory failure was defined as an oxygenation index (ratio of PaO2 (mmHg) and FiO2 (%)) ≤200 mmHg

*CPR* cardiopulmonary resuscitation, *CAD* coronary artery disease, *OHCA* out-of-hospital cardiac arrest, *IHCA* in-hospital cardiac arrest, *VT* ventricular tachycardia, *VF* ventricular fibrillation, *PEA* pulseless electrical activity, *ROSC* return of spontaneous circulation, *PCI* percutaneous coronary intervention, *N/A *not applicable

Both groups had comparable prevalence of preexisting medical conditions such as chronic heart failure, peripheral artery disease, pulmonary hypertension, and chronic liver, renal, or pulmonary disease. The cardiovascular risk profile of patients with CAD indicated greater prevalence of dyslipidemia in the CAD group (68 % vs. 31 % in the resuscitation group; *p* = 0.007) (Table 1).

Among the study population 80 % had experienced OHCA and 20 % of the study population were successfully resuscitated from IHCA. Ventricular fibrillation and ventricular tachycardia were the most common initial rhythm presentations after cardiac arrest (57 %), while 43 % of the resuscitated patients had asystole or pulseless electrical activity. The mean duration of CPR was 29.8 ± 19.1 minutes and the no-flow time was 2.4 ± 3.9 minutes. The sequential organ failure assessment (SOFA) score was calculated daily in the first 3 days after cardiac arrest and did not differ between the three measuring points in patients after CPR (Table 1).

Mean time from ROSC to blood sampling was 6.5 ± 2.9 h for the first, 25.2 ± 3.1 h for the second, and 48.8 ± 3.0 h for the third specimen of blood. A summary of routine laboratory values is shown in a supplementary table (Additional file 2).

### Monocyte TLR and inflammasome mRNA expression in patients after cardiopulmonary resuscitation and the control group

**Fig. 1:**
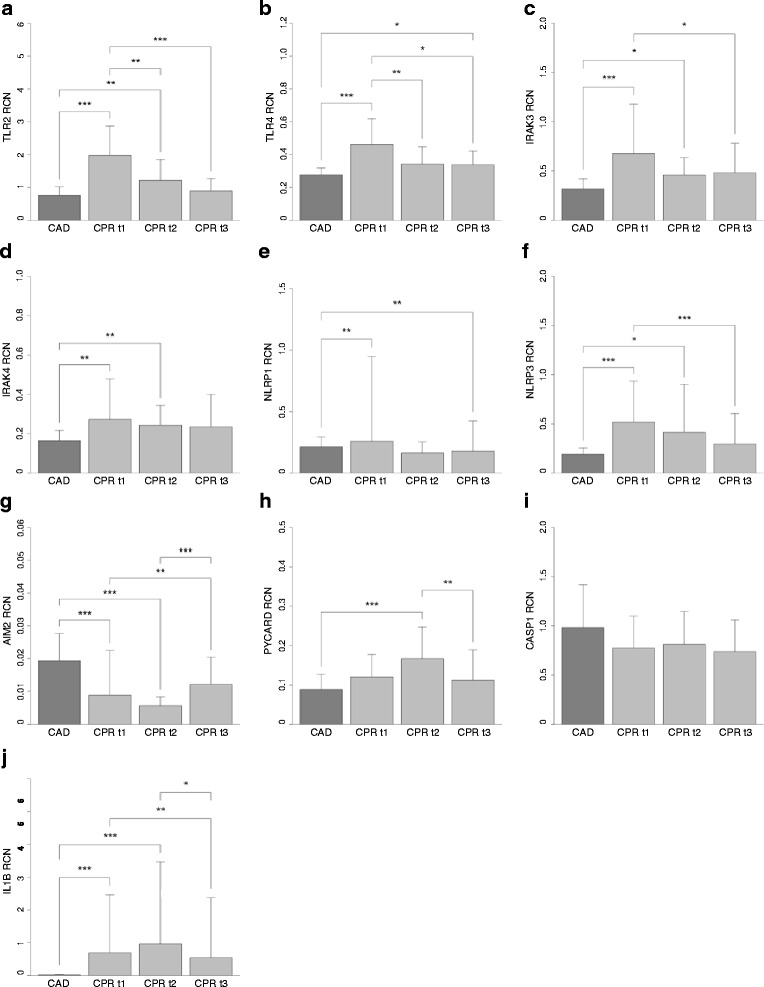
Monocyte toll-like receptor (*TLR*) and inflammasome mRNA expression in patients who had experienced cardiac arrest and the control group. Shown are monocyte mRNA expression levels of TLR2 (**a**), TLR4 (**b**), interleukin-1 receptor-associated kinase (IRAK)3 (**c**), IRAK4 (**d**), NLR family pyrin domain containing (NLRP)1 (**e**), NLRP3 (**f**), absent in melanoma (AIM)2 (**g**), PYD and CARD domain containing (PYCARD) (**h**), caspase 1 (CASP1) (**i**), and IL1B (**j**), expressed as mean relative copy numbers (*RCN*) and standard deviation, from patients after cardiopulmonary resuscitation (CPR) in the first 12 h (CPR t1; *n* = 30), after 24 h (CPR t2; *n* = 29) and 48 h (CPR t3; *n* = 23) following return of spontaneous circulation, and mRNA expression levels in the control group (coronary artery disease (CAD); *n* = 19). Statistical hypothesis testing was performed using the Kruskal-Wallis test and post-hoc analysis with all-pairwise comparison using the Dunn-Bonferroni approach (*p ≤ 0.05; **p ≤ 0.01; ***p ≤ 0.001).

In order to evaluate the potential role of PRRs in the immunoinflammatory syndrome following cardiac arrest, monocyte mRNA levels of genes related to TLR and inflammasome signaling were assessed in patients after CPR and the control group with CAD. Monocyte mRNA levels, expressed as relative copy numbers, are depicted in Fig. 1 and listed in a supplementary table (Additional file 3).

#### TLR signaling

Compared to the control group, we observed significant upregulation of surface PRR TLR2 in the early phase after cardiac arrest, which was subsequently downregulated in the later phase. Resuscitated patients had significantly higher mRNA levels of the surface PRR TLR4 in the first 12 h and 48 h after CPR with a trend towards higher levels in the intermediate phase. Likewise, IRAK4, the main kinase to further promote TLR signaling activation, was upregulated in patients in the early phase after cardiac arrest. Consistent with these results, significantly higher levels of IL1B mRNA could be detected in monocytes in the early phase after cardiac arrest. Conversely, we also observed significantly higher mRNA levels of IRAK3, a negative regulator of monocyte TLR signaling, in the first 24 h after CPR. We went on to investigate whether intracellular PRRs displayed similar regulation after CPR.

#### Inflammasome signaling

We detected distinct expression patterns of the investigated PRRs, with significant upregulation of monocyte NLRP3 mRNA levels in the first and the second blood sampling after cardiac arrest. In contrast, monocyte mRNA expression of the NLRP1 inflammasome was significantly downregulated compared to the control group in the first 12 h and at 48 h after ROSC. Likewise, we observed significantly lower mRNA expression levels of AIM2 in the first 24 h after CPR. Monocyte mRNA levels of the adaptor protein PYCARD were significantly upregulated at 24 h after ROSC. We did not observe a change in monocyte CASP1 mRNA expression in patients who had experienced cardiac arrest compared to patients with CAD.

#### Kinetics of TLR and inflammasome mRNA expression levels in the time course after cardiac arrest

In analysis of time-dependent expression of monocyte mRNA in patients after cardiac arrest, significantly higher levels of TLR2, TLR4, IRAK3, NLRP3, and IL1B were observed in patients in the early hours after ROSC compared to the later phase. In contrast, we noticed significant downregulation of AIM2 in monocytes from patients during the first 24 hours after CPR. Following the notion that these expressional changes could correlate with and possibly affect the clinical course of our patients, we performed subgroup comparison in survivors at 30 days after CPR and nonsurvivors.

### Comparison of TLR and inflammasome signaling mRNA expression levels in survivors and nonsurvivors after cardiac arrest

Interestingly, a time-dependent decrease in monocyte TLR2, TLR4, IRAK3, IRAK4, NLRP1, NLRP3, PYCARD, and IL1B mRNA expression levels was solely observed in those who did not survive for 30 days after CPR, whereas survivors had stable expression of these transcripts during the observation period. In contrast, both 30-day survivors and nonsurvivors had a time-dependent increase in monocyte AIM2 mRNA expression levels (Additional files 4 and 5).

**Fig. 2:**
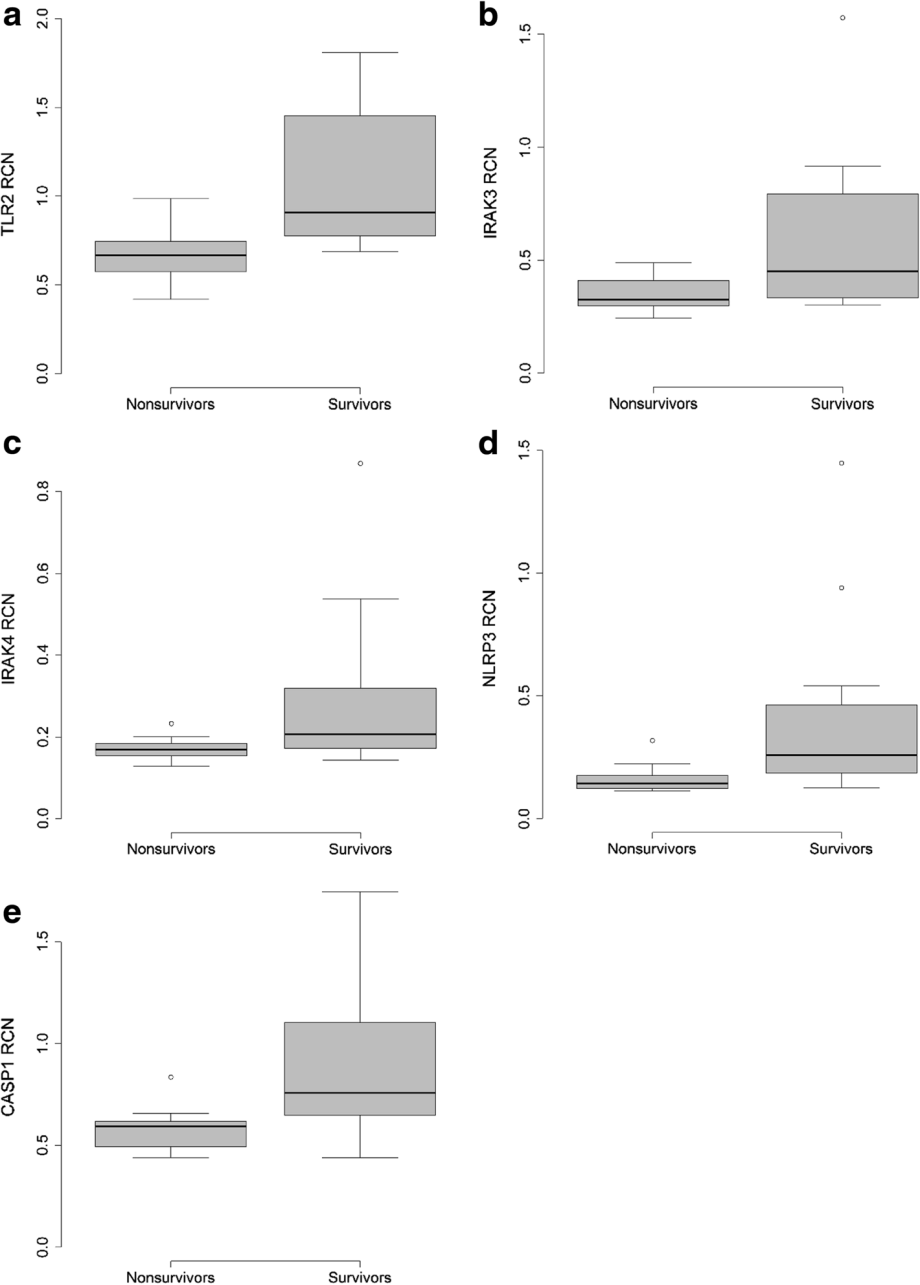
Monocyte toll-like receptor (*TLR*)2, interleukin-1 receptor-associated kinase (*IRAK*)3, IRAK4, NLR family pyrin domain containing (*NLRP*)3, and caspase (*CASP*)1 mRNA expression of survivors and nonsurvivors after cardiac arrest. Monocyte mRNA expression levels of TLR2 (**a**), IRAK3 (**b**), IRAK4 (**c**), NLRP3 (**d**), and CASP1 (**e**) in 30-day survivors (*n* = 12) and nonsurvivors (*n* = 11) after 48 h after return of spontaneous circulation (ROSC). The 30-day nonsurvivors had significantly lower monocyte TLR2 (*p* = 0.003), IRAK3 (*p* = 0.027), IRAK4 (*p* = 0.027), NLRP3 (*p* = 0.006), and CASP1 (*p* = 0.019) mRNA levels after 48 h after ROSC (*p* = 0.003). Statistical hypothesis testing was performed using the Mann–Whitney *U* test. *RCN* relative copy number

To further evaluate the prognostic implications of the investigated gene transcripts in patients who had undergone CPR, monocyte mRNA transcript levels were quantitatively compared between 30-day survivors and nonsurvivors: nonsurvivors had a trend towards higher monocyte mRNA expression levels of TLR signaling pathways in the first 12 h after ROSC, which was not statistically significant. We did not observe differences in change in monocyte TLR and inflammasome mRNA expression in CPR survivors and nonsurvivors 24 h after ROSC. Notably, we observed that 30-day nonsurvivors had significantly lower mRNA expression levels of TLR2 (*p* = 0.003), IRAK3 (*p* = 0.027), IRAK4 (*p* = 0.027), NLRP3 (*p* = 0.006), and CASP1 (*p* = 0.019) 48 h after ROSC (Table 2; Fig. 2).

**Table 2:** Monocyte mRNA expression in 30-day survivors and nonsurvivors after sudden cardiac arrest

mRNA RCN ± SD	CPR t1		*P* value	CPR t2		*P* value	CPR t3		*P* value
	Survivors (*n* = 11)	Nonsurvivors (*n* = 18)		Survivors (*n* = 11)	Nonsurvivors (*n* = 18)		Survivors (*n* = 12)	Nonsurvivors (*n* = 11)	
TLR2	1.69 ± 1.00	2.16 ± 0.84	0.061	1.32 ± 0.92	1.15 ± 0.40	0.947	1.09 ± 0.41	0.68 ± 0.17	0.003
TLR4	0.40 ± 0.15	0.51 ± 0.15	0.055	0.32 ± 0.06	0.35 ± 0.12	0.740	0.35 ± 0.09	0.33 ± 0.08	0.525
IRAK3	0.70 ± 0.80	0.67 ± 0.23	0.055	0.44 ± 0.17	0.47 ± 0.18	0.611	0.61 ± 0.37	0.35 ± 0.08	0.027
IRAK4	0.29 ± 0.32	0.26 ± 0.07	0.089	0.26 ± 0.12	0.24 ± 0.09	0.521	0.30 ± 0.21	0.17 ± 0.03	0.027
NLRP1	0.48 ± 1.14	0.13 ± 0.06	0.774	0.20 ± 0.12	0.14 ± 0.06	0.134	0.26 ± 0.32	0.10 ± 0.03	0.059
NLRP3	0.53 ± 0.59	0.51 ± 0.30	0.412	0.42 ± 0.44	0.41 ± 0.52	0.877	0.42 ± 0.40	0.16 ± 0.06	0.006
AIM2	0.015 ± 0.02	0.006 ± 0.003	0.220	0.006 ± 0.003	0.006 ± 0.003	0.774	0.012 ± 0.006	0.013 ± 0.010	0.880
PYCARD	0.11 ± 0.07	0.13 ± 0.05	0.112	0.16 ± 0.05	0.17 ± 0.10	0.550	0.14 ± 0.10	0.09 ± 0.03	0.059
CASP1	0.81 ± 0.40	0.78 ± 0.24	0.912	0.82 ± 0.17	0.80 ± 0.41	0.363	0.88 ± 0.39	0.58 ± 0.11	0.019
IL1B	1.03 ± 2.67	0.50 ± 1.03	0.877	1.25 ± 3.12	0.79 ± 2.13	0.465	1.01 ± 2.49	0.04 ± 0.07	0.118

Shown are monocyte mRNA expression levels, expressed as mean relative copy numbers (RCN) ± standard deviation (SD), in 30-day survivors and nonsurvivors in the first 12 h (cardiopulmonary resuscitation (CPR) t1; *n* = 29), after 24 h (CPR t2; *n* = 29), and 48 h (CPR t3; *n* = 23) after CPR. There was one patient in group CPR t1 who was lost to follow-up after study enrollment. Statistical hypothesis testing was performed using the Mann–Whitney *U* test. *TLR* toll-like receptor, *IRAK* interleukin-1 receptor-associated kinase, *NLRP* NLR family pyrin domain containing, *AIM* absent in melanoma, *PYCARD* PYD and CARD domain containing, *CASP *caspase

### Association of TLR signaling transcript levels and clinical markers of ischemic injury

As host-derived DAMPs from injured cells have been shown to propagate inflammation via PRRs, we hypothesized that the extent of transcriptional activation of the investigated genes of TLR and inflammasome signaling would be related to clinical markers of ischemic injury such as time from collapse to initiation of CPR, time to ROSC, serum lactate levels, and necessity of a vasopressor therapy. Correlation analyses are listed in a supplementary table (Additional file 6).

**Fig. 3:**
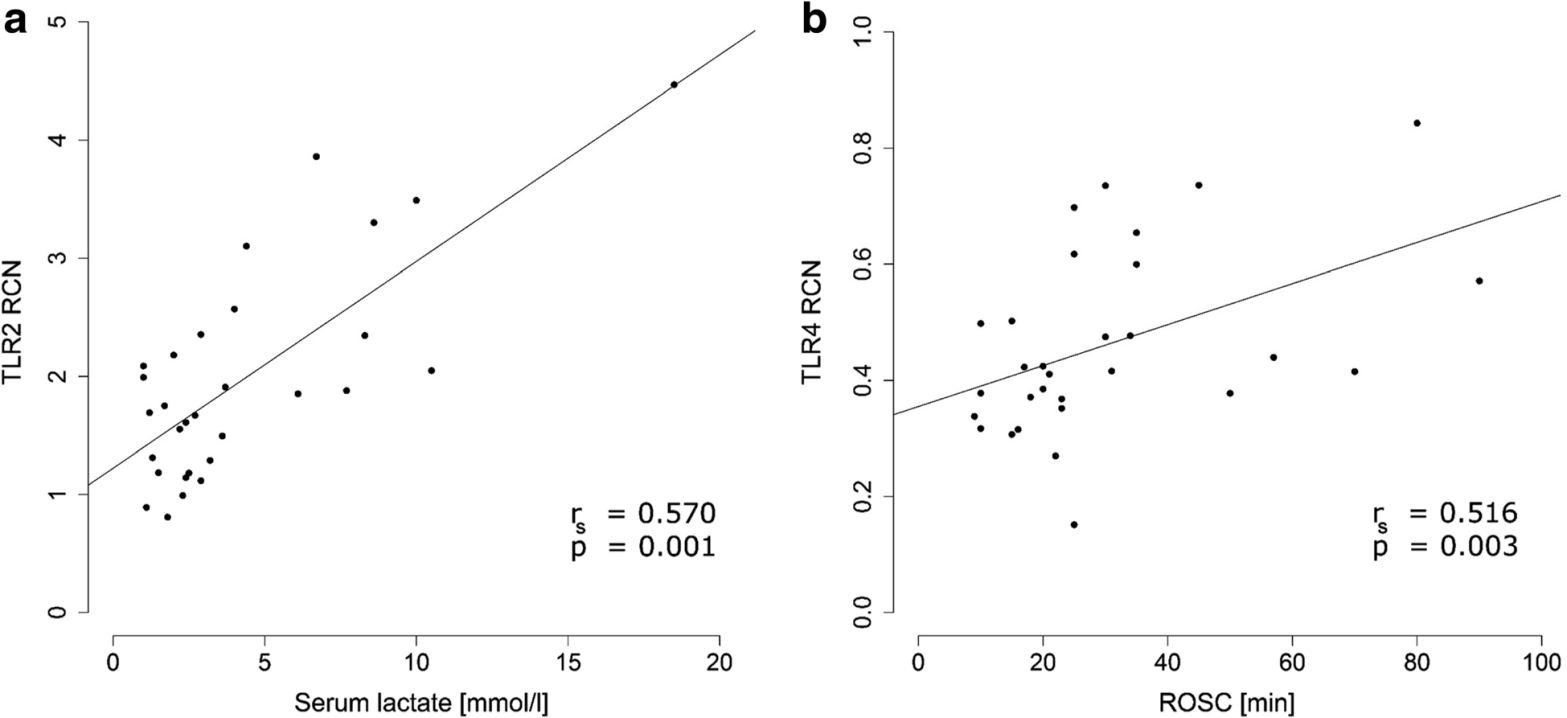
Correlation between monocyte toll-like receptor (*TLR*) expression and markers of ischemia. There was statistically significant positive correlation between serum lactate and monocyte TLR2 mRNA expression in the first 12 h after cardiac arrest (cardiopulmonary resuscitation (*CPR*) t1: *n* = 30; *r*_s_ = 0.570, *p* = 0.001) (**a**). Monocyte TLR4 mRNA levels in the first 12 h after CPR (CPR t1: *n* = 30) were positively correlated with the estimated time from the patient’s collapse until return of spontaneous circulation (*ROSC*) (*r*_s_ = 0.516, *p* = 0.003) (**b**). Statistical hypothesis testing was performed using Spearman’s rank correlation. *RCN* relative copy number

Serum lactate levels at the time of blood sampling were significantly positively correlated with TLR2 (*r*_s_ 0.570; *p* = 0.001), TLR4 (*r*_s_ 0.369; *p* = 0.045), IRAK3 (*r*_s_ 0.569; *p* = 0.001), and IRAK4 (*r*_s_ 0.413; *p* = 0.029) monocyte mRNA levels in the early phase after cardiac arrest (Fig. 3a). Monocyte NLRP1 mRNA expression was significantly negatively correlated with serum lactate levels at 24 h post CPR (*r*_s_ −0.378; *p* = 0.047). Time to ROSC was significantly positively correlated with both TLR4 (*r*_s_ 0.516; *p* = 0.003) and IRAK4 (*r*_s_ 0.407; *p* = 0.032) monocyte mRNA expression levels within the first hours after ROSC (Fig. 3b). Monocyte TLR mRNA expression was not related to estimated no-flow time from collapse to initiation of CPR or to the serum lactate directly measured after ROSC.

We observed significant positive correlation between both TLR2 and IRAK4 mRNA transcript levels and dosage of norepinephrine to maintain a mean arterial blood pressure ≥80 mmHg in the early phase after cardiac arrest.

Following the hypothesis that the observed changes in TLR and inflammasome expression are of functional relevance for the innate immune response after CPR, we investigated the functional capacity of PRR signaling in the time course following cardiac arrest by stimulating whole blood and monocytes cultures with TLR2 and TLR4 agonists.

### Proinflammatory cytokine production of cultured whole blood and monocytes in response to PRR activation

**Fig. 4:**
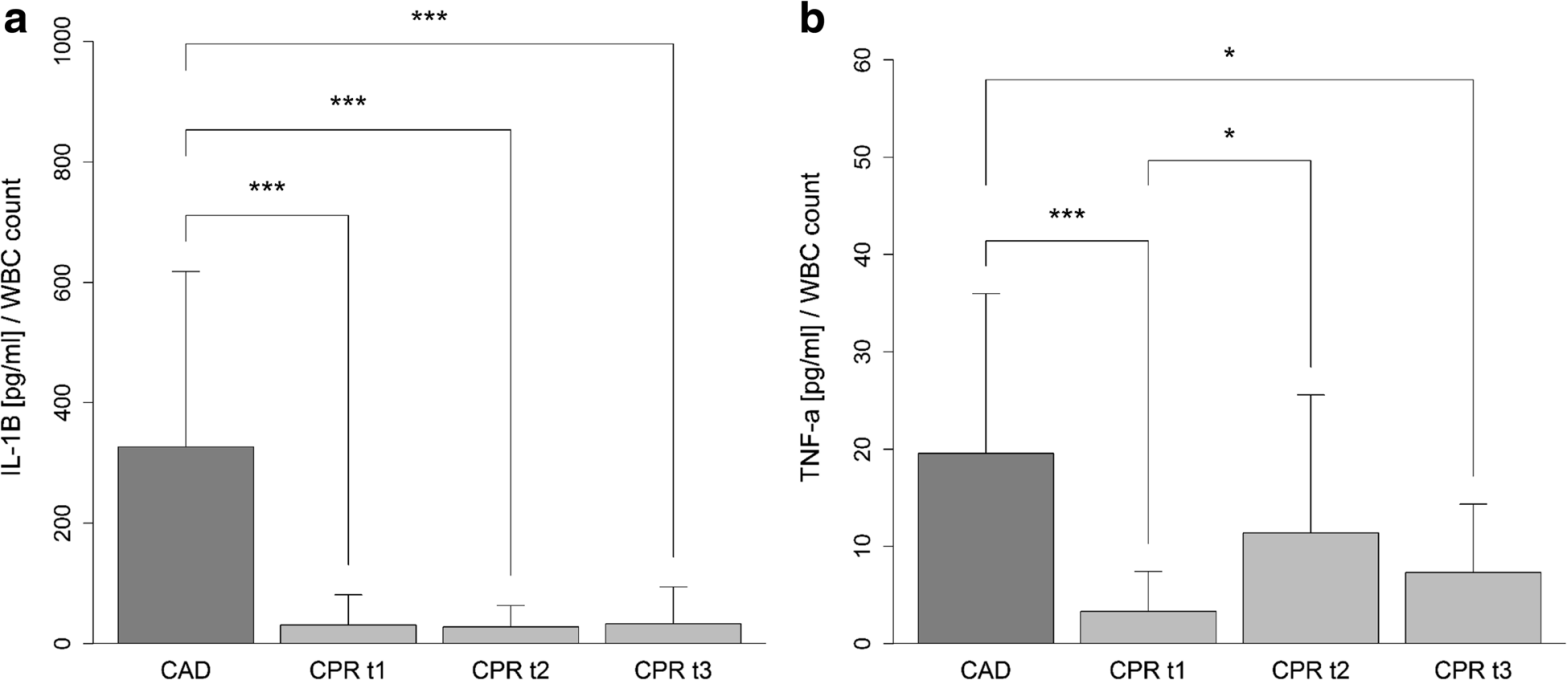
Cytokine production in whole blood in response to stimulation with toll-like receptor (TLR)2 and TLR4 agonists. Impaired IL-1β production after lipopolysaccharide stimulation of whole blood samples taken from patients in the first 12 h (cardiopulmonary resuscitation (*CPR*) t1: *n* = 19), after 24 h (CPR t2: *n* = 19), and after 48 h (CPR t3: *n* = 14) following ROSC, compared to whole blood samples from patients with coronary artery disease (*CAD*: *n* = 19) (**a**). Impaired TNF-α production after stimulation with Pam_3_CSK_4_ of whole blood samples taken from patients in the first 12 h (CPR t1: *n* = 21), after 24 h (CPR t2: *n* = 22), and after 48 h (CPR t3: *n* = 15) after CPR, compared to whole blood samples from patients with CAD (*n* = 19) (**b**). The resulting cytokine concentrations were standardized to the patient’s white blood cell count. Statistical hypothesis testing was performed using the Kruskal–Wallis test and post-hoc analysis with all-pairwise comparison using the Dunn–Bonferroni approach (*p value ≤0.05; ***p value ≤0.001)

**Fig. 5:**
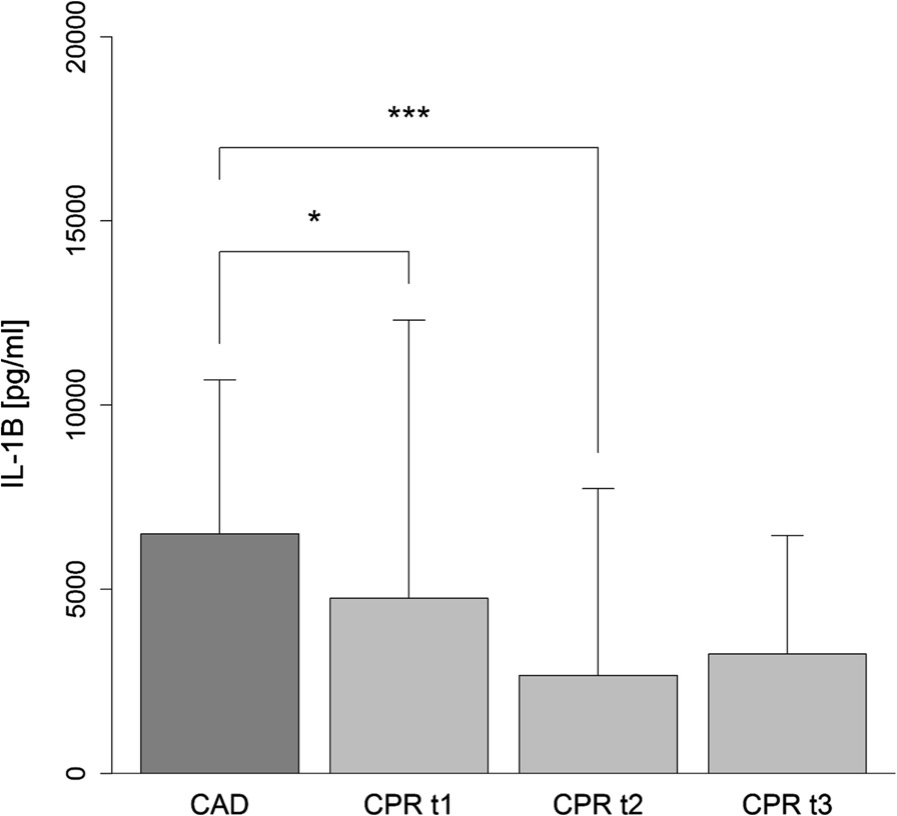
Cytokine production of cultured monocytes in response to stimulation with lipopolysaccharide (LPS). IL-1β production of LPS-stimulated monocyte cultures from resuscitated patients in the first 12 h (cardiopulmonary resuscitation (*CPR*) t1: n = 25), after 24 h (CPR t2: n = 28), and after 48 h (CPR t3: n = 19) following return of spontaneous circulation, and from patients with coronary artery disease (*CAD*: n = 19). Statistical hypothesis testing was performed using the Kruskal–Wallis test and post-hoc analysis with all-pairwise comparison using the Dunn–Bonferroni approach (*p value ≤0.05; ***p value ≤0.001)

Whole blood samples taken from patients after cardiac arrest had markedly impaired synthesis of IL-1β in response to stimulation with the TLR4 agonist LPS (CAD 327.0 ± 291.3; CPR t1 31.3 ± 49.7; CPR t2 28.0 ± 35.6; CPR t3 33.5 ± 60.6 ((pg/ml)/white blood cell (WBC) count)). Similarly, there was less TNF-α production induced in Pam_3_CSK_4_-activated whole blood samples taken from patients after cardiac arrest. This effect was more pronounced in the early phase following ROSC (CAD 19.6 ± 16.4; CPR t1 3.3 ± 4.1; CPR t2 11.4 ± 14.2; CPR t3 7.3 ± 7.0 ((pg/ml)/WBC count)) (Fig. 4). Interestingly, cultured monocytes also had impaired IL-1β production in response to stimulation with LPS in the first 24 h after CPR (CAD 6514.7 ± 4178.8; CPR t1 4764.4 ± 7550.5; CPR t2 2662.8 ± 5064.8; CPR t3 3243.5 ± 3224.5 ((pg/ml)) (Fig. 5). To investigate if these observed differences in cytokine production are mediated by humoral factors in the serum of the resuscitated patients, we performed in vitro serum exchange experiments.

### Proinflammatory cytokine production of cultured PBMCs after serum exchange in vitro

Stimulation of cultured PBMCs from a healthy volunteer with patient serum at a concentration of 20 % did not induce a detectable amount of IL-1β in most culture supernatants, with very low and comparable concentrations in the samples where detection was possible (Additional file 7). Interestingly, cultured PBMCs from a healthy individual had an attenuated inflammatory response to LPS after co-stimulation with serum from resuscitated patients compared to co-stimulation with serum from the control group (CAD 1298.57 ± 370.15; CPR t1 698.57 ± 645.09; CPR t3 650 ± 338.08 ((pg/ml)) (Additional file 8).

## Discussion

In this study, we provide evidence for the activation of TLR2 and TLR4 in immediate survivors of cardiac arrest and describe the involvement of the NLRP3 inflammasome in the modulation of the subsequent systemic inflammatory response to global IRI caused by temporary circulatory arrest. Our findings suggest the innate immune system as a possible pathophysiological factor in PCAS and as a potential therapeutic target for the treatment of this condition.

PCAS is characterized by a global IRI that results in significant inflammation in multiple organs, which leads to both mortality due to organ failure and morbidity due to neurological impairment in eventual survivors. Our own group has recently shown that different populations of proinflammatory microparticles [34] and a perturbation of the endothelial glycocalyx [11] likely contribute to initiating the early phases of PCAS. Others have reported a significant increase in inflammatory cytokines and their receptors during the course of the PCAS, including IL-1ra, IL-6, IL-8, IL-10 and sTNFRII [8]. However, the molecular events that govern this systemic reaction to circulatory arrest and finally result in activation of inflammatory cells remain little understood.

### TLR expression

Upregulation of TLR2 and TLR4 in response to the presence of PAMPs has extensively been studied in non-sterile inflammatory conditions like sepsis and septic shock [27, 35–37]. However, there is a growing body of evidence that TLR2 and TLR4 also play a pivotal role in sterile inflammatory conditions such as acute and chronic cardiovascular diseases [20, 38–40]. We detected temporary upregulation of monocyte TLR2, TLR4, and IRAK4, the main kinase to further propagate TLR signaling, immediately after ROSC, which possibly resembles the strong inflammatory activation induced by the global IRI. Accordingly, there was a significant positive correlation of these markers with the degree of ischemic injury as assessed by serum lactate levels, the duration of CPR, and the dosage of norepinephrine to sustain adequate blood pressure. However, there was no correlation between TLR signaling mRNA expression levels and the initial serum lactate measured directly after ROSC, indicating that failure of lactate clearance and persisting tissue hypoxia might be relevant for the regulation of TLR signaling in whole body IRI. This interpretation is further supported by recent findings from Selejan and coworkers who demonstrated upregulation of TLR2 in patients with cardiogenic shock and correlation of TLR2 expression with the “symptom to reperfusion time” [40]. Interestingly, the initial upregulation was followed by relative downregulation of both TLR2 and TLR4 at later time points, which was more evident in 30-day nonsurvivors. This finding is in good correspondence with similar observations during the time course of sepsis [41], coronary artery bypass grafting [42], and percutaneous coronary intervention [31], and possibly contributes to the development of a compensatory anti-inflammatory response syndrome (CARS), an adapted response to dampen the overzealous inflammatory response [43].

A key mechanism for the development of CARS is thought to be a phenomenon called endotoxin tolerance (ET), a transient state in which monocytes and macrophages are unable to respond to endotoxin [44], which has been extensively studied in sepsis [45, 46]. This mechanism is thought to be partly mediated by downregulation of surface TLR4 expression [47]. However, TLR expression and ex vivo cytokine release were not correlated in our study, indicating the involvement of regulatory adaptor molecules in the functional TLR response. IRAK3 has been shown to negatively regulate downstream TLR signaling [48] and to mediate LPS tolerance in human models of endotoxemia [49]. Indeed, we detected early upregulation of the negative regulator of TLR signaling IRAK3 in our patient population at a time point when TLR expression was still high, but TLR response was already attenuated.

### Inflammasome expression

Processing and release of IL-1β is regulated on multiple levels. Activation of surface PRRs, such as TLR4, lead to generation of inactive pro IL-1β; activation and assembly of the inflammasome, a cytoplasmatic multiprotein complex that consists of a sensor protein (e.g., NLRP1, NLRP3, or AIM2), the adaptor protein PYCARD, and the protease caspase-1, then leads to proteolytic cleavage of pro-IL-1β into its biologically active form IL-1β. A large variety of exogenous and endogenous danger signals, including extracellular ATP, uric acid crystals, and potassium influx, have been shown to activate the inflammasome [22, 50, 51]. In our patient population, we now detected a distinct regulation of the different inflammasomes in isolated circulating monocytes, with significant upregulation of the NLRP3 inflammasome in the first 24 h after CPR and downregulation of the NLRP1 and the AIM2 inflammasome. The latter finding is in good correspondence with a recent investigation in patients with septic shock [27], where a similar downregulation was observed compared to critically ill patients and healthy controls. One possible explanation of this differential expression of the inflammasome subsets could be their ligand specificity: while NLRP3 is activated by a large number of pathogens and intrinsic stimuli, NLRP1 is predominantly described in the innate immune response to microbial pathogens [52], which represents a secondary process in PCAS following the initial sterile inflammation. Similar to our findings on TLR expression, the upregulation of NLRP3 was restricted to the early phase after cardiac arrest. Both NLRP3 and CASP1 mRNA expression levels were significantly lower in patients who died compared to eventual survivors. Furthermore, we observed a trend towards downregulation of NLRP1 and PYCARD in nonsurvivors. This is in line with findings determining NLRP1 as an independent predictor of mortality in patients with septic shock [27]. How this phenomenon represents a normal physiological effect to limit excessive inflammation, or a maladaptive response that predisposes the organism to secondary infections, remains unclear.

### Monocyte inflammatory response

On a functional level, we observed a pronounced and sustained decrease in inflammatory cytokine release after TLR2 and TLR4 activation in patients after cardiac arrest ex vivo. Interestingly, sera from resuscitated patients attenuated the inflammatory response of PBMCs from a healthy volunteer after stimulation with LPS. This phenomenon known as endotoxin tolerance or TLR hyporesponsiveness is well-documented and can be observed in both endotoxin-dependent settings, such as sepsis [45, 46], and endotoxin-independent settings, such as major trauma [53] and vascular surgery [42]. Our findings are in good correspondence with a study by Adrie and coworkers who were the first to investigate the immunoinflammatory profile of patients after successful CPR and who demonstrated the development of ET in these patients [8]. Our findings are further supported by a recent study from Beurskens and coworkers, who compared plasma cytokine levels and the TLR response to LPS and lipoteichoic acid in patients after cardiac arrest [54].

Mechanistic analyses of ET in genetically modified mice have suggested the differential regulation of TLR-adaptor proteins as a causal factor for this phenomenon [55]. In our study we observed upregulation of the pseudokinase IRAK3. IRAK3 belongs to the IL-1 receptor-associated kinase family and serves as a negative regulator downstream of TLR4. Induction of IRAK3 is associated with LPS-induced ET in humans [49]. Furthermore, mice deficient in IRAK3 are known not to display ET in vivo [48]. Similar regulation of IRAK3, as demonstrated in this study, was previously described in sepsis [56] and myocardial infarction [57], suggesting that upregulation of IRAK3 could be a common mechanism of ET across these different pathological conditions.

Our experimental findings fit the hypothesis that patients undergo a whole body IRI after cardiac arrest [58], with a release of DAMPs, which finally leads to PRR activation and ET after subsequent stimulation. Our group has previously reported the presence of DAMPs in patients after cardiac arrest, which are known to be endogenous TLR and inflammasome ligands [11, 34]. Accordingly, a recent study from Timmermans and coworkers demonstrated significant associations between the presence of DAMPs in survivors of cardiac arrest and the intensity of ET in the first days after CPR [59]. However non-sterile activation of PRRs , also has to be taken in account because endotoxemia [16] and bacteremia [60] have been reported in resuscitated patients and gastric aspiration is a common event after CPR [16]. Our current study corroborates the hypothesis that ET is mediated by both soluble serum factors and intrinsic leucocyte reprogramming [8] and expands these findings to a larger patient population. In addition, it identifies an important cell population for this phenotypic response and contributes to the mechanistic explanation of ET by demonstrating the differential regulation of the involved receptors and cytosolic modulators of the monocyte response to PRR activation.

### Study limitations

As with all clinical studies in the field of cardiac arrest research, the definition of an appropriate control population is difficult. We decided on patients with coronary artery disease, as most patients in our CPR group had circulatory arrest of cardiac origin and received similar pharmacological and interventional treatment to the control group. However, the control population was not subjected to therapeutic hypothermia, which could result in a significant confounder, as cooling can potentially attenuate the IRI [61]. As all resuscitated patients were treated with mild therapeutic hypothermia (expect one patient who died before the target temperature was reached), no analysis of the effects of cooling within this group was possible. However, our serial measurements in the individual patients are not affected by this bias and from a pathophysiological point of view the cooling should result in underestimation of the observed inflammation in the CPR group. Furthermore, in the study by Beurskens et al., leucocyte cytokine release was not affected by body temperature [54].

As we focused on monocytes as a specific circulating cell population, potential divergent effects in other resting or circulating cell types therefore remain beyond the scope of this study. As the amount of blood that could be sampled from the critically ill patients was limited, our analysis of the isolated monocytes was limited to RNA expression levels and measurements of individual cytokines at the protein level. As inflammasome activation is controlled by both fast-acting post-translational mechanisms and slower-acting transcriptional regulation, our PCR-based analysis can only describe changes due to the latter mechanism [62].

Finally, due to inherent limitations of an observational study, the causal relationship between our findings and the development of the PCAS cannot be deducted from our study. Also, our sample size was limited to 51 patients who had undergone CPR at a single institution.

## Conclusions

With the lack of effective treatment options after cardiac arrest, the clarification of the underlying pathophysiology of the PCAS is a prerequisite for future therapy. Theoretically, intrinsic DAMPs and the interaction with their receptor could represent attractive therapeutic targets in this setting, as these molecules are only released during injury. Several inhibitors of different components of innate immune signaling are currently under development and a humanized anti-TLR2 antibody was recently shown to decrease myocardial IRI in pigs [63], whereas a specific TLR-4 inhibitor exhibited similar effects in IRI of the brain [64]. The notion that TLR2 might exhibit an important role in PCAS is further supported by a recent study where the administration of a TLR2 inhibiting antibody or genetic TLR2 deficiency improved survival and neurological function in mice after circulatory arrest [65]. However, potential unwanted attenuation of the host defense against infection has to be taken into account with these strategies.

Our findings directly demonstrate the differential regulation of monocyte TLR expression and function in immediate survivors of cardiac arrest and implicate the NLRP3 inflammasome as a potential downstream mediator of the inflammatory response during PCAS. The time course of monocyte inflammatory marker expression and function suggests a proinflammatory phenotype in the early phase after ROSC and compensating suppression of monocyte-mediated inflammation during the progress of the syndrome. How far these findings functionally determine the progression of PCAS remains to be determined in future interventional studies, but modulation of the innate immune response by targeted therapies has the theoretical potential to attenuate global IRI in the early phase of PCAS and septic inflammatory complications in the later phase.

## Key messages

- Monocyte TLR2, TLR4 and NLRP3 inflammasome signaling is differentially regulated in the time course of PCAS.
- Patients who do not survive after cardiac arrest have decreased expression of monocyte TLR2, IRAK3, IRAK4, NLRP3 and CASP1 in the later time course of PCAS.
- Patients who undergo CPR exhibit profound endotoxin tolerance ex vivo, which is possibly mediated in an IRAK3-dependent manner.

## Abbreviations

ANOVA, analysis of variance; AIM, absent in melanoma; β2M, beta-2 microglobulin; CAD, coronary artery disease; CARS, compensatory anti-inflammatory response syndrome; CASP, caspase; CPR, cardiopulmonary resuscitation; DAMP, danger-associated molecular pattern; DPSB, Dulbecco’s phosphate buffered saline; ET, endotoxin tolerance; IHCA, in-hospital cardiac arrest; IL-1β, interleukin-1 beta (protein); IL1B, interleukin-1 beta (gene); IRI, ischemia-reperfusion injury; LPS, lipopolysaccharide; OHCA, out-of-hospital cardiac arrest; IRAK, interleukin-1 receptor-associated kinase; NLRP, NLR family pyrin domain containing; PAD, peripheral artery disease; PAMP, pathogen-associated molecular pattern; PBMC, peripheral blood mononuclear cell; PCAS, post-cardiac-arrest syndrome; PCI, percutaneous coronary intervention; PEA, pulseless electrical activity; POL2RA, RNA polymerase 2; PRR, pattern recognition receptor; PYCARD, PYD and CARD domain containing; qPCR, quantitative real-time polymerase chain reaction; RCN, relative copy number; ROSC, return of spontaneous circulation; RPMI, Roswell Park Memorial Institute; SD, standard deviation; SOFA, sequential organ failure assessment; TLR, toll-like receptor; TNFα, tumor necrosis factor alpha; VF, ventricular fibrillation; VT, ventricular tachycardia; WBC, white blood cell.

## Additional files

Additional file 1: Primer list. Accession number = Refseq accession number, web page access date 3 July 2014 (http://www.ncbi.nlm.nih.gov/refseq/). *TA* annealing temperature, *Conc.* concentration of each primer pair, *Amplicon* length of replicated DNA sequence in base pairs (bp). (DOCX 16 kb) Additional file 2: Laboratory tests. Shown are patients’ inflammatory laboratory tests at admission, 24 and 48 h after ROSC. *Laboratory tests from patients after cardiopulmonary resuscitation (*CPR*) versus coronary artery disease (*CAD*) at admission. ^†^Laboratory tests from patients after CPR versus CPR at admission. (DOCX 15 kb) Additional file 3: Monocyte mRNA expression in patients who had suffered cardiac arrest and the control group. Shown are kinetics of monocyte mRNA expression levels, expressed as mean relative copy numbers ± standard deviation (SD), in patients who had suffered cardiac arrest in the first 12 h (*CPR t1*; *n* = 30), after 24 h (CPR t2; *n* = 29) and after 48 h (CPR t3; *n* = 23) following CPR, and in the control group with coronary artery disease (*CAD*; *n* = 19). Statistical hypothesis testing was performed using the Kruskal–Wallis test and post-hoc analysis with all-pairwise comparison using the Dunn–Bonferroni approach indicated as the *p* values listed above. (DOCX 15 kb) Additional file 4: Time-dependent monocyte mRNA expression in 30-day nonsurvivors following cardiac arrest. Shown are monocyte mRNA expression levels of TLR2, TLR4, IRAK3, IRAK4. NLRP1, NLRP3, AIM2, PYCARD, CASP1, and IL-1β in 30-day nonsurvivors in the first 12 h (CPR t1: *n* = 18), after 24 h (CPR t2: *n* = 18), and after 48 h (CPR t3: *n* = 11) following ROSC. Statistical hypothesis testing was performed using the Kruskal–Wallis test and post-hoc analysis with all-pairwise comparison using the Dunn–Bonferroni approach (**p* value ≤0.05; ***p* value ≤0.01; ****p* value ≤0.001). (DOCX 42 kb) Additional file 5: Time-dependent monocyte mRNA expression in 30-day survivors following cardiac arrest. Shown are monocyte mRNA expression levels of TLR2, TLR4, IRAK3, IRAK4. NLRP1, NLRP3, AIM2, PYCARD, CASP1, and IL-1β in 30-day survivors in the first 12 h (CPR t1: *n* = 11), after 24 h (CPR t2: *n* = 11), and after 48 h (CPR t3: *n* = 12) following ROSC. Statistical hypothesis testing was performed using the Kruskal–Wallis test and post-hoc analysis with all-pairwise comparison using the Dunn–Bonferroni approach (**p* value ≤0.05). (DOCX 36 kb) Additional file 6: Correlation analyses of monocyte mRNA expression levels and clinical characteristics. Shown are correlation analyses of monocyte mRNA expression levels from patients in the first 12 h (CPR t1; *n* = 30), after 24 h (CPR t2; *n* = 29), and after 48 h (CPR t3; *n* = 23) following CPR, and the corresponding clinical characteristics. There was one patient lost to follow up after study enrollment. Statistical hypothesis testing was performed using Spearman’s rank correlation indicated as Spearman’s rho (*r*_s_) and the *p* values listed above. *CPR* cardiopulmonary resuscitation, *ROSC* return of spontaneous circulation, *lactate* serum lactate; *t0* at admission, *NE* dosage of norepinephrine to maintain mean arterial blood pressure ≥80 mmHg. (DOCX 16 kb) Additional file 7: Cytokine production of cultured PBMCs in response to stimulation with patients’ sera. Shown is interleukin-1β (IL-1β) production of cultured PBMCs from a healthy volunteer in response to stimulation with 20 % serum either from patients with coronary artery disease (CAD: *n* = 8) or from resuscitated patients in the first 12 h (CPR t1: *n* = 14) and after 48 h following cardiac arrest (CPR t3: *n* = 9). Production of IL-1β did not statistically differ between the three groups. Statistical hypothesis testing was performed using the Kruskal–Wallis test. (DOCX 32 kb) Additional file 8: Cytokine production of cultured PBMCs in response to co-stimulation with patients’ sera and LPS. Shown is interleukin-1β (IL-1β) production of cultured PBMCs from a healthy volunteer in response to co-stimulation with 10 ng/ml LPS and 20 % serum either from patients with coronary artery disease (CAD: *n* = 7) or from resuscitated patients in the first 12 h (CPR t1: *n* = 14) and after 48 h following cardiac arrest (CPR t3: *n* = 9). Statistical hypothesis testing was performed using one-way ANOVA and post-hoc analysis with all-pairwise comparison using the Games-Howell approach (**p* value ≤0.05; ***p* value ≤0.01). (DOCX 41 kb)
