# Supplementary material for: Inflammasome and toll-like receptor signaling in human monocytes after successful cardiopulmonary resuscitation
Source: Crit Care. 2016 Jun 4;20:170. doi: 10.1186/s13054-016-1340-3 (PMC4893227; doi:10.1186/s13054-016-1340-3)
Supplement: Additional file 1: — Primer list. Accession number = Refseq accession number, web page access date 3 July 2014 (http://www.ncbi.nlm.nih.gov/refseq/). TA annealing temperature, Conc. concentration of each primer pair, Amplicon length of replicated DNA sequence in base pairs (bp). (DOCX 16 kb) [file 13054_2016_1340_MOESM1_ESM.docx]

**Additional file 1: Primer list**

| Primer | Accession number | Primer sequence | T_A_ | Conc. | Properties | Amplicon |
| --- | --- | --- | --- | --- | --- | --- |
| POLR2A | NM_000937.4 | Sense: GCACCACGTCCAATGACAT Antisense: GTGCGGCTGCTTCCATAA | 60 °C | 0.5 µM 0.5 µM | intron-flanking | 267 bp |
| B2M | NM_004048.2 | Sense: CTATCCAGCGTACTCCAA Antisense: CCAGACACATAGCAATTCAG | 60 °C | 0.2 µM 0.2 µM | intron-spanning | 88 bp |
| TLR2 | NM_003264.3 | Sense: CACTGGACAATGCCACATAC Antisense: GCGGTCACAAGACAGAGAAG | 60 °C | 0.5 µM 0.5 µM | within the same exon | 105 bp |
| TLR4 | NM_138554.4 | Sense: CCTGAGGCATTTAGGCAGC Antisense: CTTGATAGTCCAGAAAAGGC | 60 °C | 0.5 µM 0.5 µM | intron-flanking | 186 bp |
| IRAK3 | NM_007199.2 | Sense: GGTCATCTGTGGCAGTATA Antisense: ACAACTCTGATGTTCTAGGT | 58 °C | 0.5 µM 0.5 µM | intron-flanking | 115 bp |
| IRAK4 | NM_001114182.2 | Sense: AGTAATGGCAAAGTGTCAAC Antisense: GAGTCTGTCTAGCAATGAAC | 60 °C | 0.4 µM 0.4 µM | intron-spanning | 118 bp |
| NLRP1 | NM_033004.3 | Sense: CTTCTCCTGCCTCTCAAG Antisense: AAGTGAACTCGGTACAAGT | 60 °C | 0.5 µM 0.5 µM | intron-spanning | 127 bp |
| NLRP3 | NM_004895.4 | Sense: CTTCAGGTGTTGGAATTAGAC Antisense: GCACTTCACAGAACATCAT | 60 °C | 0.4 µM 0.4 µM | intron-spanning | 151 bp |
| AIM2 | NM_004833.1 | Sense: GTCCAGAAGTGTCAGAGT Antisense: GCCTGTTAGCAAGAGTATC | 58 °C | 0.5 µM 0.5 µM | intron-flanking | 197 bp |
| PYCARD | NM_013258.4 | Sense: CTTCTACCTGGAGACCTAC Antisense: CTTCCCGTACAGAGCATC | 60 °C | 0.4 µM 0.4 µM | intron-flanking | 244 bp |
| IL1B | NM_000576.2 | Sense: GCCCTAAACAGATGAAGTGCTCCT Antisense: CCTGAAGCCCTTGCTGTAGTG | 58 °C | 0.5 µM 0.5 µM | intron-spanning | 104 bp |
| CASP1 | NM_033292.3 | Sense: TCGGCAGAGATTTATCCA Antisense: GTTCTTCTAGGAATACTGTCAA | 58 °C | 0.5 µM 0.5 µM | intron-spanning | 95 bp |

Accession number = Refseq accession number, web page access date July 3, 2014, <http://www.ncbi.nlm.nih.gov/refseq/>
T_A_ = annealing temperature; Conc. = concentration of each primer-pair; Amplicon = length of replicated DNA sequence in base pairs (bp)
