# Supplementary material for: Inflammasome and toll-like receptor signaling in human monocytes after successful cardiopulmonary resuscitation
Source: Crit Care. 2016 Jun 4;20:170. doi: 10.1186/s13054-016-1340-3 (PMC4893227; doi:10.1186/s13054-016-1340-3)
Supplement: Additional file 2: — Laboratory tests. Shown are patients’ inflammatory laboratory tests at admission, 24 and 48 h after ROSC. *Laboratory tests from patients after cardiopulmonary resuscitation (CPR) versus coronary artery disease (CAD) at admission. †Laboratory tests from patients after CPR versus CPR at admission. (DOCX 15 kb) [file 13054_2016_1340_MOESM2_ESM.docx]

**Additional file 2:** **Laboratory tests**

|  | | | CPR group  (n = 51) |  | CAD group (n = 19) |  | p-value |
| --- | --- | --- | --- | --- | --- | --- | --- |
| Laboratory tests | | |  |  |  |  |  |
|  | At admission: | |  |  |  |  |  |
|  | | White blood count (10³/µl) | 16.03 ± 7.45 |  | 7.53 ± 2.19 |  | **0.000*** |
|  | | Platelet count (10³/µl) | 208 ± 70.36 |  | 233.58 ± 66.67 |  | 0.332* |
|  | | C-reactive protein (mg/l) | 15.24 ± 24.79 |  | 5.24 ± 4.05 |  | 0.236* |
|  | | Procalcitonin (ng/ml) | 1.50 ± 6.00 |  |  |  | N/A |
|  | 24 h after ROSC | |  |  |  |  |  |
|  | | White blood count (10³/µl) | 13.57 ± 6.64 |  |  |  | 0.089^†^ |
|  | | Platelet count (10³/µl) | 190.26 ± 69.73 |  |  |  | 0.186^†^ |
|  | | C-reactive protein (mg/l) | 39.61 ± 38.71 |  |  |  | **0.000**^†^ |
|  | | Procalcitonin (ng/ml) | 3.46 ± 6.41 |  |  |  | **0.000**^†^ |
|  | 48 h after ROSC | |  |  |  |  |  |
|  | | White blood count (10³/µl) | 12.27 ± 5.40 |  |  |  | **0.024**^†^ |
|  | | Platelet count (10³/µl) | 166.22 ± 51.50 |  |  |  | **0.001**^†^ |
|  | | C-reactive protein (mg/l) | 115.25 ± 58.58 |  |  |  | **0.000**^†^ |
|  | | Procalcitonin (ng/ml) | 3.23 ± 5.17 |  |  |  | **0.000**^†^ |

Shown are patients’ inflammatory laboratory tests at admission, as well as 24 and 48 hours following ROSC.
* = laboratory tests from patients after CPR versus CAD at admission
† = laboratory tests from patients after CPR versus CPR at admission
CPR, cardiopulmonary resuscitation; CAD, coronary artery disease
