# Supplementary material for: Inflammasome and toll-like receptor signaling in human monocytes after successful cardiopulmonary resuscitation
Source: Crit Care. 2016 Jun 4;20:170. doi: 10.1186/s13054-016-1340-3 (PMC4893227; doi:10.1186/s13054-016-1340-3)
Supplement: Additional file 3: — Monocyte mRNA expression in patients who had suffered cardiac arrest and the control group. Shown are kinetics of monocyte mRNA expression levels, expressed as mean relative copy numbers ± standard deviation (SD), in patients who had suffered cardiac arrest in the first 12 h (CPR t1; n = 30), after 24 h (CPR t2; n = 29) and after 48 h (CPR t3; n = 23) following CPR, and in the control group with coronary artery disease (CAD; n = 19). Statistical hypothesis testing was performed using the Kruskal–Wallis test and post-hoc analysis with all-pairwise comparison using the Dunn–Bonferroni approach indicated as the p values listed above. (DOCX 15 kb) [file 13054_2016_1340_MOESM3_ESM.docx]

**Additional file 3: Monocyte mRNA expression in patients after cardiac arrest and control group**

| mRNA |  | Mean RCN ± SD | |  |  |  |  | p-value | |  |  |  |  |  |
| --- | --- | --- | --- | --- | --- | --- | --- | --- | --- | --- | --- | --- | --- | --- |
|  |  | CAD  (n = 19) | CPR t1  (n = 30) | | CPR t2 (n = 29) | CPR t3 (n = 23) |  | CAD vs  CPR t1 | CAD vs  CPR t2 | | CAD vs CPR t3 | CPR t1 vs CPR t2 | CPR t1 vs CPR t3 | CPR t2 vs CPR t3 |
|  |  |  |  | |  |  |  |  |  | |  |  |  |  |
| TLR2 |  | 0.76 ± 0.26 | 1.97 ± 0.90 | | 1.22 ± 0.64 | 0.89 ± 0.38 |  | **0.000** | **0.007** | | 1.000 | **0.008** | **0.000** | 0.076 |
| TLR4 |  | 0.28 ± 0.04 | 0.46 ± 0.16 | | 0.34 ± 0.11 | 0.34 ± 0.08 |  | **0.000** | 0.083 | | **0.047** | **0.003** | **0.019** | 1.000 |
| IRAK3 |  | 0.32 ± 0.10 | 0.68 ± 0.50 | | 0.46 ± 0.18 | 0.48 ± 0.30 |  | **0.000** | **0.023** | | 0.161 | 0.076 | **0.019** | 1.000 |
| IRAK4 |  | 0.16 ± 0.05 | 0.27 ± 0.21 | | 0.24 ± 0.10 | 0.24 ± 0.16 |  | **0.002** | **0.007** | | 0.264 | 1.000 | 0.609 | 1.000 |
| PYCARD |  | 0.09 ± 0.04 | 0.12 ± 0.06 | | 0.17 ± 0.08 | 0.11 ± 0.08 |  | 0.296 | **0.000** | | 1.000 | 0.058 | 1.000 | **0.006** |
| NLRP1 |  | 0.21 ± 0.08 | 0.26 ± 0.69 | | 0.17 ± 0.09 | 0.18 ± 0.24 |  | **0.007** | 0.280 | | **0.002** | 0.945 | 1.000 | 0.313 |
| NLRP3 |  | 0.19 ± 0.06 | 0.52 ± 0.42 | | 0.42 ± 0.48 | 0.30 ± 0.31 |  | **0.000** | **0.022** | | 1.000 | 0.262 | **0.000** | 0.150 |
| AIM2 |  | 0.02 ± 0.01 | 0.01 ± 0.01 | | 0.01 ± 0.00 | 0.01 ± 0.01 |  | **0.000** | **0.000** | | 0.088 | 1.000 | **0.008** | **0.001** |
| CASP1 |  | 0.98 ± 0.43 | 0.77 ± 0.33 | | 0.81 ± 0.33 | 0.74 ± 0.32 |  | 0.436 | 1.000 | | 0.069 | 1.000 | 1.000 | 0.904 |
| IL1B |  | 0.02 ± 0.01 | 0.69 ± 1.78 | | 0.96 ± 2.51 | 0.54 ± 1.83 |  | **0.000** | **0.001** | | 1.000 | 1.000 | **0.009** | **0.027** |

Shown are kinetics of monocyte mRNA expression levels, expressed as mean relative copy numbers ± standard deviation (SD), in the first 12 hours (CPR t1; n = 30), after 24 hours (CPR t2; n = 29) and 48 hours (CPR t3; n = 23) following CPR, as well as of the control group with coronary artery disease (CAD; n = 19). Statistical hypothesis testing was performed by Kruskal Wallis test and post-hoc analysis with all-pairwise comparison using Dunn-Bonferroni approach indicated as the p-values listed above.
