# Supplementary material for: Inflammasome and toll-like receptor signaling in human monocytes after successful cardiopulmonary resuscitation
Source: Crit Care. 2016 Jun 4;20:170. doi: 10.1186/s13054-016-1340-3 (PMC4893227; doi:10.1186/s13054-016-1340-3)
Supplement: Additional file 4: — Time-dependent monocyte mRNA expression in 30-day nonsurvivors following cardiac arrest. Shown are monocyte mRNA expression levels of TLR2, TLR4, IRAK3, IRAK4. NLRP1, NLRP3, AIM2, PYCARD, CASP1, and IL-1β in 30-day nonsurvivors in the first 12 h (CPR t1: n = 18), after 24 h (CPR t2: n = 18), and after 48 h (CPR t3: n = 11) following ROSC. Statistical hypothesis testing was performed using the Kruskal–Wallis test and post-hoc analysis with all-pairwise comparison using the Dunn–Bonferroni approach (*p value ≤0.05; **p value ≤0.01; ***p value ≤0.001). (DOCX 42 kb) [file 13054_2016_1340_MOESM4_ESM.docx]

**Additional file 4: Time-dependent monocyte mRNA expression in 30-days nonsurvivors following cardiac arrest**


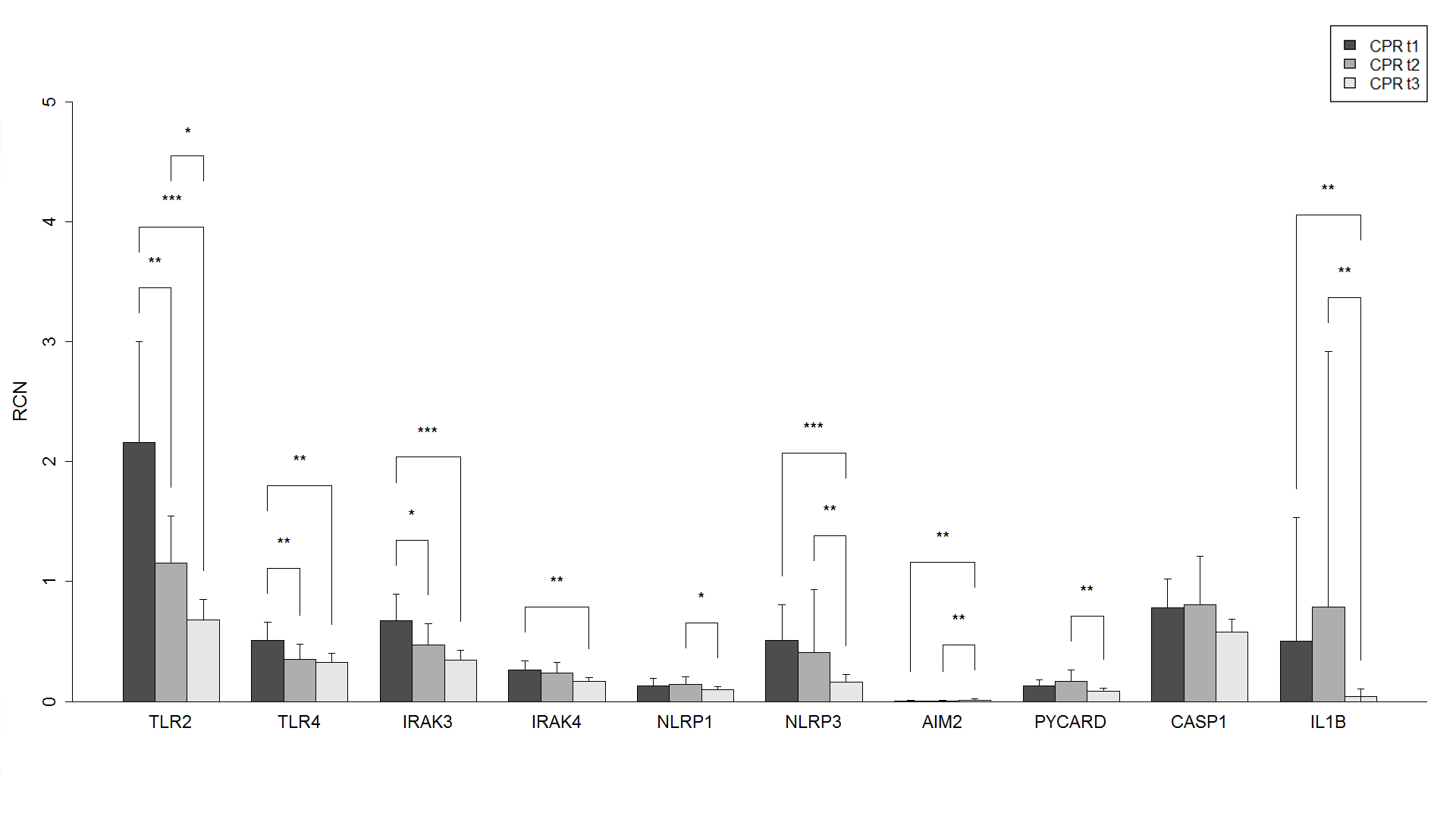

Shown are monocyte mRNA expression levels of TLR2, TLR4, IRAK3, IRAK4. NLRP1, NLRP3, AIM2, PYCARD, CASP1, and IL1B in 30-days nonsurvivors in the first 12 hours (CPR t1: n = 18), after 24 hours (CPR t2: n = 18), and 48 hours (CPR t3: n = 11) following ROSC. Statistical hypothesis testing was performed by Kruskal Wallis test and post-hoc analysis with all-pairwise comparison using Dunn-Bonferroni approach indicated as the p-values shown as asterisks (*: p-value ≤ 0.05; **: p-value ≤ 0.01; ***: p-value ≤ 0.001).
