# Supplementary material for: Inflammasome and toll-like receptor signaling in human monocytes after successful cardiopulmonary resuscitation
Source: Crit Care. 2016 Jun 4;20:170. doi: 10.1186/s13054-016-1340-3 (PMC4893227; doi:10.1186/s13054-016-1340-3)
Supplement: Additional file 6: — Correlation analyses of monocyte mRNA expression levels and clinical characteristics. Shown are correlation analyses of monocyte mRNA expression levels from patients in the first 12 h (CPR t1; n = 30), after 24 h (CPR t2; n = 29), and after 48 h (CPR t3; n = 23) following CPR, and the corresponding clinical characteristics. There was one patient lost to follow up after study enrollment. Statistical hypothesis testing was performed using Spearman’s rank correlation indicated as Spearman’s rho (r s) and the p values listed above. CPR cardiopulmonary resuscitation, ROSC return of spontaneous circulation, lactate serum lactate; t0 at admission, NE dosage of norepinephrine to maintain mean arterial blood pressure ≥80 mmHg. (DOCX 16 kb) [file 13054_2016_1340_MOESM6_ESM.docx]

**Additional file 6: Correlation analyses of monocyte mRNA expression levels and clinical characteristics**

| mRNA |  | | | Collapse to ROSC [min] | | Collapse to CPR [min] | | Lactate t0 [mM] | | Lactate t1 [mM] | | | Lactate t2 [mM] | | | Lactate t3 [mM] | | | NE t1 [mg/h] | | | NE t2 [mg/h] | | | NE t3 [mg/h] | | |  |
| --- | --- | --- | --- | --- | --- | --- | --- | --- | --- | --- | --- | --- | --- | --- | --- | --- | --- | --- | --- | --- | --- | --- | --- | --- | --- | --- | --- | --- |
| Group |  | | | CPR t1 (n = 30) | | CPR t1 (n = 30) | | CPR t1 (n = 30) | | CPR t1 (n = 30) | | | CPR t2 (n = 28) | | | CPR t3 (n = 22) | | | CPR t1 (n = 30) | | | CPR t2 (n = 29) | | | CPR t3 (n = 22) | | |  |
|  |  | |  | | |  | |  | |  | |  | | |  | | |  | | |  | | |  | |  | | |
| TLR2 | r_s_:  p: | 0.353 0.056 | | | - 0.102 0.591 | | 0.173 0.361 | | **0.570 0.001** | | 0.152 0.439 | | | 0.189 0.400 | | | **0.413 0.023** | | | - 0.100 0.607 | | | 0.141 0.533 | | | |  | |
| TLR4 | r_s_: p: | **0.516 0.003** | | | - 0.136 0.473 | | 0.219 0.246 | | **0.369 0.045** | | 0.269 0.166 | | | 0.190 0.389 | | | 0.320 0.085 | | | - 0.007 0.973 | | | - 0.046 0.840 | | | |  | |
| IRAK3 | r_s_: p: | 0.213 0.259 | | | - 0.043 0.823 | | 0.110 0.564 | | **0.569 0.001** | | 0.363 0.058 | | | 0.307 0.164 | | | 0.297 0.111 | | | 0.116 0.550 | | | 0.336 0.126 | | | |  | |
| IRAK4 | r_s_: p: | **0.407 0.032** | | | - 0.167 0.395 | | 0.034 0.863 | | **0.413 0.029** | | - 0.048 0.807 | | | 0.307 0.164 | | | **0.388 0.041** | | | 0.055 0.776 | | | 0.160 0.477 | | | |  | |
| PYCARD | r_s_: p: | 0.142 0.455 | | | - 0.025 0.896 | | - 0.077 0.684 | | 0.287 0.124 | | - 0.060 0.763 | | | 0.156 0.489 | | | 0.094 0.622 | | | 0.063 0.745 | | | - 0.076 0.736 | | | |  | |
| NLRP1 | r_s_: p: | - 0.221 0.240 | | | - 0.171 0.366 | | - 0.328  0.077 | | - 0.015 0.936 | | **- 0.378 0.047** | | | 0.248 0.266 | | | - 0.204  0.279 | | | - 0.191 0.322 | | | 0.216 0.334 | | | |  | |
| NLRP3 | r_s_: p: | 0.264 0.159 | | | - 0.187 0.322 | | 0.015  0.937 | | 0.322 0.083 | | 0.276 0.155 | | | 0.066 0.770 | | | 0.156 0.412 | | | - 0.070 0.717 | | | - 0.168 0.456 | | | |  | |
| AIM2 | r_s_: p: | 0.337 0.069 | | | - 0.079 0.679 | | - 0.111 0.558 | | - 0.019 0.920 | | - 0.175 0.374 | | | - 0.235 0.293 | | | 0.294 0.115 | | | 0.050 0.795 | | | - 0.100 0.658 | | | |  | |
| CASP1 | r_s_: p: | 0.260 0.165 | | | - 0.081 0.669 | | - 0.136 0.472 | | 0.189 0.317 | | 0.060 0.763 | | | - 0.132 0.557 | | | 0.285 0.127 | | | 0.078 0.688 | | | - 0.237 0.289 | | | |  | |
| IL1B | r_s_: p: | 0.306 0.100 | | | - 0.269 0.150 | | - 0.013 0.946 | | 0.280 0.133 | | 0.140 0.478 | | | - 0.115 0.611 | | | 0.252 0.180 | | | 0.003 0.989 | | | - 0.208 0.352 | | | |  | |

Shown are correlation analyses of monocyte mRNA expression levels from patients in the first 12 hours (CPR t1; n = 30), after 24 hours (CPR t2; n = 29), and 48 hours (CPR t3; n = 23) following CPR and corresponding clinical characteristics. There was one patient with loss of follow-up after study enrollment. Statistical hypothesis testing was performed by Spearman’s rank correlation indicated as Spearman’s rho (rs) and the p-values listed above.
CPR, cardiopulmonary resuscitation; ROSC, return of spontaneous circulation, lactate, serum lactate; t0, at admission; NE = dosage of norepinephrine to maintain a mean arterial blood pressure ≥ 80 mmHg
