# Supplementary material for: Inflammasome and toll-like receptor signaling in human monocytes after successful cardiopulmonary resuscitation
Source: Crit Care. 2016 Jun 4;20:170. doi: 10.1186/s13054-016-1340-3 (PMC4893227; doi:10.1186/s13054-016-1340-3)
Supplement: Additional file 7: — Cytokine production of cultured PBMCs in response to stimulation with patients’ sera. Shown is interleukin-1β (IL-1β) production of cultured PBMCs from a healthy volunteer in response to stimulation with 20 % serum either from patients with coronary artery disease (CAD: n = 8) or from resuscitated patients in the first 12 h (CPR t1: n = 14) and after 48 h following cardiac arrest (CPR t3: n = 9). Production of IL-1β did not statistically differ between the three groups. Statistical hypothesis testing was performed using the Kruskal–Wallis test. (DOCX 32 kb) [file 13054_2016_1340_MOESM7_ESM.docx]

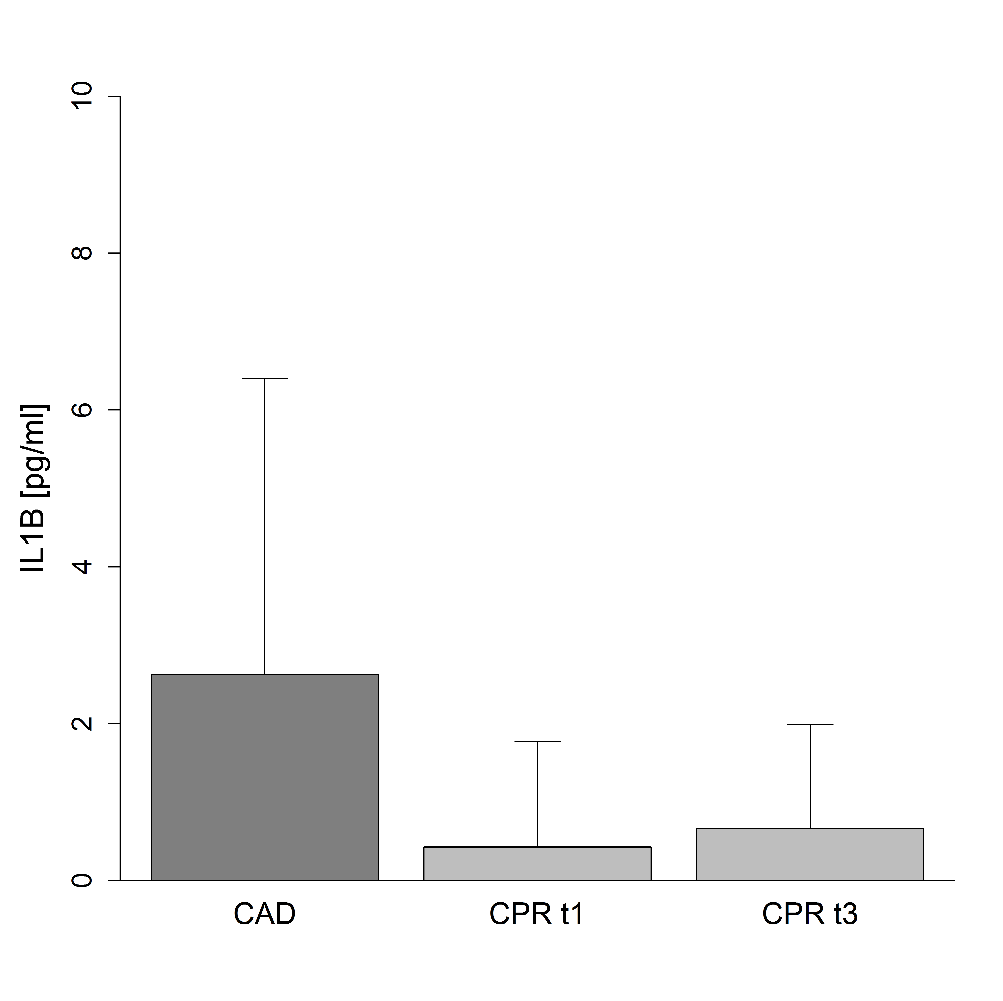
**Additional file 7:** **Cytokine production of cultured PBMCs in response to stimulation with patients’ sera**

Shown is interleukin-1β (IL1B) production of cultured PBMCs from a healthy volunteer in response to stimulation with 20% serum from either patients with coronary artery disease (CAD: n = 8) or resuscitated patients in the first 12 hours (CPR t1: n = 14) and after 48 hours following cardiac arrest (CPR t3: n = 9). Production of IL1B did not statistically differ between the three groups. Statistical hypothesis testing was performed by Kruskal-Wallis test.
