# Supplementary material for: Inflammasome and toll-like receptor signaling in human monocytes after successful cardiopulmonary resuscitation
Source: Crit Care. 2016 Jun 4;20:170. doi: 10.1186/s13054-016-1340-3 (PMC4893227; doi:10.1186/s13054-016-1340-3)
Supplement: Additional file 8: — Cytokine production of cultured PBMCs in response to co-stimulation with patients’ sera and LPS. Shown is interleukin-1β (IL-1β) production of cultured PBMCs from a healthy volunteer in response to co-stimulation with 10 ng/ml LPS and 20 % serum either from patients with coronary artery disease (CAD: n = 7) or from resuscitated patients in the first 12 h (CPR t1: n = 14) and after 48 h following cardiac arrest (CPR t3: n = 9). Statistical hypothesis testing was performed using one-way ANOVA and post-hoc analysis with all-pairwise comparison using the Games-Howell approach (*p value ≤0.05; **p value ≤0.01). (DOCX 41 kb) [file 13054_2016_1340_MOESM8_ESM.docx]

**
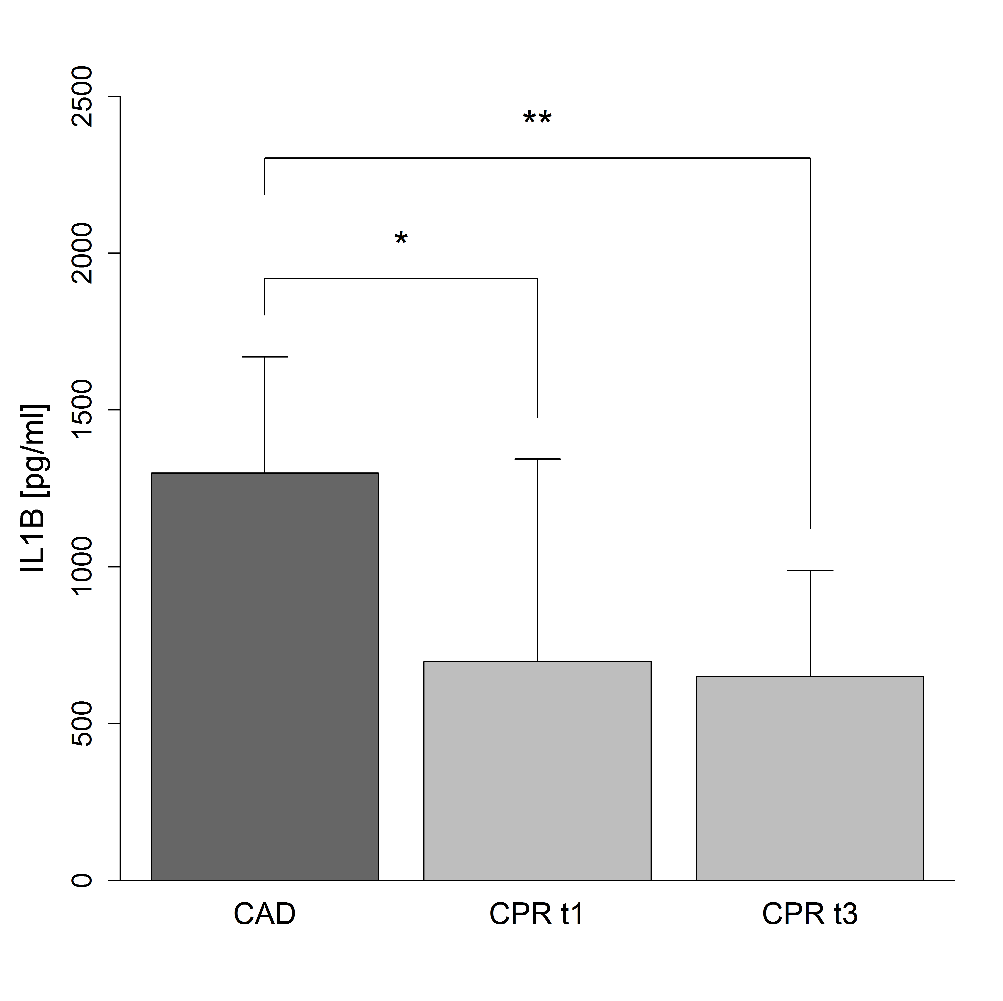
Additional file 8: Cytokine production of cultured PBMCs in response to co-stimulation with patients’ sera and LPS.**

Shown is interleukin-1β (IL1B) production of cultured PBMCs from a healthy volunteer in response to co-stimulation with 10 ng/ml LPS and 20% serum from either patients with coronary artery disease (CAD: n = 7) or resuscitated patients in the first 12 hours (CPR t1: n = 14) and after 48 hours following cardiac arrest (CPR t3: n = 9). Statistical hypothesis testing was performed by one-way ANOVA and post-hoc analysis with all-pairwise comparison using Games-Howell approach indicated as the p-values shown as asterisks (*: p-value ≤ 0.05; **: p-value ≤ 0.01).
